# Crystal structure of bis­(μ-*N*-hy­droxy­picolin­amid­ato)bis­[bis­(*N*-hy­droxy­picolinamide)­sodium]

**DOI:** 10.1107/S2056989016019095

**Published:** 2017-01-01

**Authors:** Inna S. Safyanova, Kateryna A. Ohui, Irina V. Omelchenko

**Affiliations:** a Department of Chemistry, National Taras Shevchenko University of Kyiv, Volodymyrska Street 64, 01601 Kiev, Ukraine; b SSI "Institute for Single Crystals", National Academy of Sciences of Ukraine, Nauki ave. 60, Kharkiv, 61001, Ukraine

**Keywords:** crystal structure, hydroxamic acids, hydrogen bonds, π–π stacking

## Abstract

The title compound, [Na_2_(C_6_H_5_N_2_O_2_)_2_(C_6_H_6_N_2_O_2_)_4_], is a centrosymmetric coordination dimer based on the sodium(I) salt of *N*-hy­droxy­picolinamide. The mol­ecule has an {Na_2_O_6_(μ-O)_2_} core with two bridging carbonyl O atoms and two hydroxamate O atoms of two mono-deprotonated residues of *N*-hy­droxy­picolinamide, while two neutral *N*-hy­droxy­picolinamide mol­ecules are coordinated in a monodentate manner to each sodium ion *via* the carbonyl O atoms [the Na—O distances range from 2.3044 (2) to 2.3716 (2) Å]. The penta­coordinated sodium ion exhibits a distorted trigonal–pyramidal coordination polyhedron. In the crystal, the coordination dimers are linked into chains along the *c* axis *via* N—H⋯O and N—H⋯N hydrogen bonds; the chains are linked into a two-dimensional framework parallel to (100) *via* weak C—H⋯O and π–π stacking inter­actions.

## Abstract 2

In the crystal, the coordination dimers are linked *via* N—H⋯O, N—H⋯N and C—H⋯O hydrogen bonds and π–π stacking inter­actions into a two-dimensional framework parallel to (100).

## Chemical context   

Hydroxamic acids as a class of organic compounds originate from Lossen’s invention (Lossen, 1869 ▸). The coordination ability of hydroxamic acids has led to their extensive use in coordination and supra­molecular chemistry (Świątek-Kozłowska *et al.*, 2000 ▸; Dobosz *et al.*, 1999 ▸). In particular, over the past two decades they have often been used as frameworks of metallacrowns (Golenya *et al.*, 2012*a* ▸; Safyanova *et al.*, 2015 ▸; Stemmler *et al.*, 1999 ▸; Jankolovits *et al.*, 2013*a* ▸,*b* ▸) and as building blocks of coordination polymers (Gumienna-Kontecka *et al.*, 2007 ▸; Golenya *et al.*, 2014 ▸; Pavlishchuk *et al.*, 2010 ▸, 2011 ▸). They have also been studied intensively in biology and medicine due to their various biological activities, especially their metal-chelating ability and inhibition of a series of metalloenzymes (Codd, 2008 ▸; Griffith *et al.*, 2005 ▸; Marmion *et al.*, 2013 ▸).
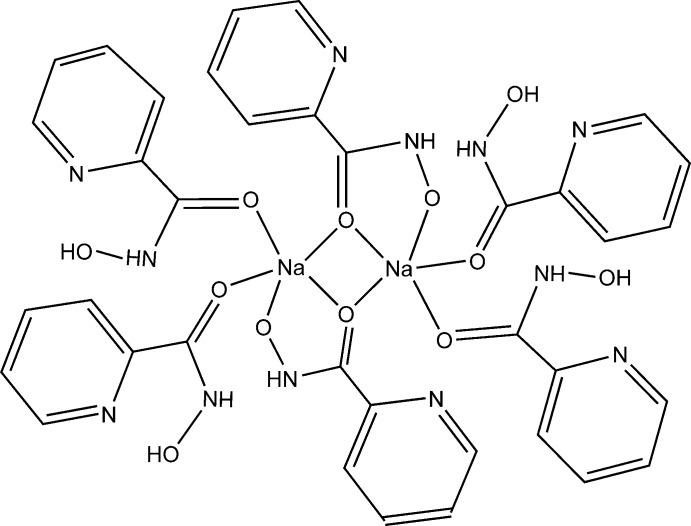


*N*-Hy­droxy­picolinamide (or picoline-2-hydroxamic acid, H_2_PicHA) has been used extensively for the synthesis of polynuclear complexes, especially various metallacrowns (Stemmler *et al.*, 1999 ▸; Seda *et al.*, 2007 ▸; Jankolovits *et al.*, 2013*a* ▸; Golenya *et al.*, 2012*a* ▸; Gumienna-Kontecka *et al.*, 2013 ▸). A large number of polynuclear metal complexes based on this ligand has been investigated. The Cambridge Structural Database (Groom *et al.*, 2016 ▸) contains data on the crystal structures of over 20 coordination compounds based on *o*-PicHA. The crystal and mol­ecular structure of *N*-hy­droxy­picolinamide monohydrate was the subject of two recent independent investigations (Chaiyaveij *et al.*, 2015 ▸; Safyanova *et al.*, 2016 ▸).

In the course of the synthesis of hydroxamate metal complexes, especially metallacrowns, in some cases alkaline metal hydroxamates appear to be more preferable starting materials than the parent hydroxamic acids due to their better solubility in water. During our synthetic attempts using the sodium salt of *N*-hy­droxy­picolinamide, we noticed that the elementary analysis data differ noticeably from those expected for the monosodium salt or its hydrates, which might affect the reagent ratio in the synthesis of coordination compounds. In order to find out the reason for this deviation in the analytical data, we undertook a single crystal X-ray analysis of the sodium salt of *N*-hy­droxy­picolinamide. Herein we present the crystal and mol­ecular structure of the title compound.

## Structural commentary   

**Figure 1:**
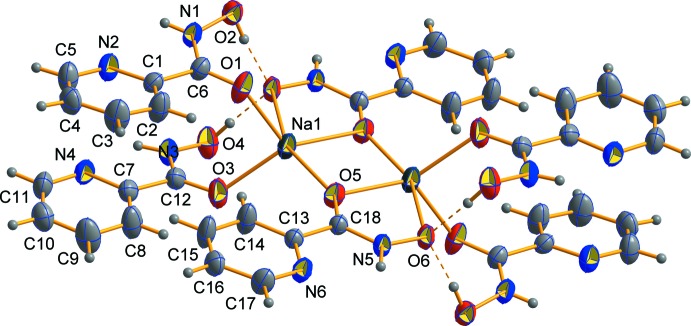
The centrosymmetric molecular unit of the title compound, with displacement ellipsoids drawn at the 50% probability level. H atoms are shown as spheres of undefined radius.

The mol­ecular structure of title compound is shown in Fig. 1 ▸. The structure determination revealed that the dinuclear hydroxamate acid salt was obtained, with the ratio of neutral and deprotonated *N*-hy­droxy­picolinamide being 2:1. A centrosymmetric dimeric structure is formed by non-planar subunits inter­connected through the bridging carbonyl O atoms belonging to the deprotonated residues of *N*-hy­droxy­picolinamide [the Na—μ-O distances are Na1—O5 = 2.3044 (14) Å and Na1—O5^i^ = 2.3558 (14) Å; symmetry code: (i) 1 − *x*, −*y*, 1 − *z*] . Coordination of the μ-O carbonyl and hydroxamate O atoms of the same anion lead to the formation of five-membered chelate rings [Na1—O6^i^ = 2.3716 (14) Å and O5^i^—Na—O6^i^ = 70.26 (5)°]. Two neutral *N*-hy­droxy­picolinamide mol­ecules coordinate in a monodentate manner to each sodium ion *via* the carbonyl O atoms [Na1—O1 = 2.3300 (16) Å and Na1—O3 = 2.3225 (15) Å]. As a result, each penta­coordinated sodium ion reveals a distorted trigonal–pyramidal coordination polyhedron (τ_5_ = 0.50; Addison *et al.*, 1984 ▸) with O1, O3, and O5^i^ atoms forming the equatorial plane. The distance between the equatorial plane and the Na atom is 0.408 (1) Å and the deviation of the O—Na—O angles from ideal values are up to 23.47 (5)°. The Na—O bond lengths are in the range 2.3044 (14)—2.3716 (14) Å, which is common for penta­coordinated sodium cations (Groom *et al.*, 2016 ▸; Golenya *et al.*, 2012*b* ▸; Malinkin *et al.*, 2012*a* ▸,*b* ▸). The central Na_2_(μ-O)_2_ core is virtually planar and approaches a square [the O—Na—O angles are 86.43 (5) and 93.57 (5)°].

The deprotonated hydroxamate atom O6 acts as an acceptor of two hydrogen bonds (Table 1 ▸) in which the O—H groups of the protonated hydroxamic groups of two neutral mol­ecules of *N*-hy­droxy­picolinamide act as donors [O2—H2⋯O6(1 − *x*, −*y*, 1 − *z*) = 1.65 (2) Å and 169 (2)°; O4—H4⋯O6(1 − *x*, −*y*, 1 − *z*) = 1.66 (3) Å and 177 (3)°]. The nearly coplanar pyridine rings of two neutral mol­ecules of *N*-hy­droxy­picolinamide coordinating to the same sodium ion reveal intra­molecular stacking inter­actions in unusual ‘head-to-head’ manner [angle between planes = 10.00 (7)°, inter­centroid distance = 3.801 (1) Å, mean inter­planar separation = 3.760 (1) Å, mean plane shift = 0.508 (4) Å].

The deprotonated *N*-hy­droxy­picolinamide residue adopts a strongly flattened conformation with a dihedral angle of only 0.6 (2)° between the hydroxamic group and the pyridine ring. At the same time, the corresponding dihedral angles in both neutral *N*-hy­droxy­picolinamide mol­ecules are noticeably greater [17.5 (2) and 8.9 (2)°], indicating a deviation of the hydroxamic group from the plane of pyridine rings. The configuration about the hydroxamic C—N bond is *Z* and that about the C—C bond between the pyridine and hydroxamic groups is *E* for both the neutral and deprotonated hydroxamates. Intra­molecular N—H⋯N attractive contacts between the hydroxamate group and the nitro­gen atom of pyridine ring [2.25 (2)–2.35 (3) Å] are present in both the neutral and deprotonated *N*-hy­droxy­picolinamide mol­ecules (Table 1 ▸).

The bond lengths and angles within both the neutral and deprotonated hydroxamic groups are within normal ranges. The C—N and C—C bond lengths in the pyridine moiety are typical for 2-substituted pyridine derivatives (Moroz *et al.*, 2012 ▸; Strotmeyer *et al.*, 2003 ▸; Fritsky *et al.*, 2004 ▸).

## Supra­molecular features   

**Figure 2:**
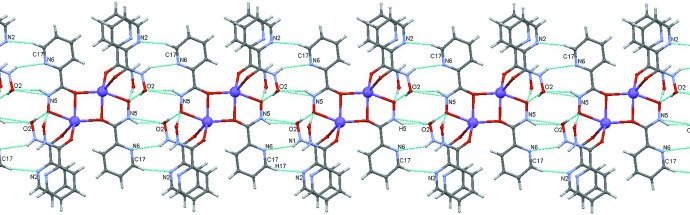
A packing diagram of the title compound. Hydrogen bonds are indicated by dashed lines. **[The outline of the unit cell and axes should be added]**

In the crystal (Fig. 2 ▸), the dimeric mol­ecules are linked into chains along the *c* axis *via* two pairs of classical inter­molecular N5—H5⋯O2(*x*, *y*, *z* − 1) and N1—H1⋯N6(*x*, *y*, *z* − 1) hydrogen bonds supported by a pair of weak non-classical C17—H17⋯N2(*x*, *y*, *z* − 1) hydrogen bonds (Table 1 ▸). The chains are linked into a two-dimensional framework parallel to (100) by weaker inter­actions, namely a C5—H5*A*⋯O4(−*x* + 1, −*y* + 1, −*z* + 2) hydrogen bond and π–π stacking between the N4/C7–C11 pyridine ring and the deprotonated O5/C18/N5/O6 hydroxamic group [angle between planes = 4.89 (7)°, inter­centroid distance = 3.766 (1) Å, mean inter­planar separation = 3.385 (2) Å, mean plane shift = 1.644 (4) Å]. Inter­molecular π–π stacking between the same deprotonated hydroxamic group and the N2/C1–C5 pyridine ring [angle between planes = 10.78 (8)°, inter­centroid distance = 3.823 (1) Å, mean inter­planar separation = 3.589 (2) Å, mean plane shift = 1.319 (4) Å] links the frameworks into a three-dimensional structure.

## Database survey   

A search of the Cambridge Structural Database (Groom *et al.*, 2016 ▸) for metal complexes based on *N*-hy­droxy­picolinamide revealed the crystal structures of over 20 compounds, mostly belonging to the metallacrown (MC) family. In particular, heterometallic copper(II) 15-metallacrown-5 complexes with encapsulated Gd^III^ and Eu^III^ ions (Stemmler *et al.*, 1999 ▸), Ca^2+^, Pr^3+^ and Nd^3+^ ions (Safyanova *et al.*, 2015 ▸), UO_2_^2+^ (Stemmler *et al.*, 1996 ▸), and Pb^2+^ and Hg^2+^ ions (Seda *et al.*, 2007 ▸; Saf’yanova *et al.*, 2014 ▸) have been structurally characterized. Nickel(II) 15-metallacrown-5 complexes with Eu^3+^ (Jankolovits *et al.*, 2013*b* ▸), Sm^3+^ and Pb^2+^ ions (Seda *et al.*, 2006*a* ▸) in the central cavity have also been synthesized and structurally characterized. Homo-[12-MC_Zn(II),picHA_-4](OTf)_1.25_(OH)_0.75_ (Jankolovits *et al.*, 2013*a* ▸) and heterometallic zinc(II) 12-metallacrown-4 complexes including sandwich compounds Dy^III^[12-MC_Zn(II),picHA_-4]_2_(OH)_3_(py)_2_ (Jankolovits *et al.*, 2014 ▸) and Tb^III^[12-MC_Zn(II),picHA_-4]_2_·[24 MC_Zn(II),picHA_-8]·(pyridine)_8_·(triflate)_3_ (Jankolovits *et al.*, 2011 ▸) have also been reported. Three structures of collapsed copper(II) metallacrowns have been reported (Golenya *et al.*, 2012*a* ▸) as well as a trinuclear mixed-ligand copper(II) complex with pyridine (Seda *et al.*, 2006*b* ▸) and 2,2′-di­pyridine (Gumienna-Kontecka *et al.*, 2013 ▸), and mono- and binuclear complexes with platinum(II) (Griffith *et al.*, 2005 ▸). In addition, a tetra­nuclear Zn_4_(picHA)_2_(OAc)_4_(DMF)_2_ collapsed metallacrown complex has been struct­urally characterized (Jankolovits *et al.*, 2013*a* ▸).

## Synthesis and crystallization   

The title compound was obtained by the reaction of *N*-hy­droxy­picolinamide (0.156 g, 1 mmol, dissolved in 5 ml of water) with sodium hydrogen carbonate (1 *M* aqueous solution, 1 ml). Colorless crystals suitable for X-ray diffraction were obtained from the resulting aqueous solution by slow evaporation at ambient temperature within 48 h (yield 78%).

## Refinement   

Crystal data, data collection and structure refinement details are summarized in Table 2 ▸. All hydrogen atoms were found in the difference Fourier maps; H atoms of pyridine rings were constrained to ride on their parent atoms with C—H = 0.93 Å and *U*_iso_ = 1.2*U*_eq_(C), and H atoms of the N—H and O—H groups were refined isotropically.

## Supplementary Material

Crystal structure: contains datablock(s) I, global. DOI: 10.1107/S2056989016019095/xu5895sup1.cif

Structure factors: contains datablock(s) I. DOI: 10.1107/S2056989016019095/xu5895Isup2.hkl

CCDC reference: 1520114

Additional supporting information: crystallographic information; 3D view; checkCIF report

## Figures and Tables

**Table 1:** Hydrogen-bond geometry (Å, °)

*D*—H⋯*A*	*D*—H	H⋯*A*	*D*⋯*A*	*D*—H⋯*A*
N1—H1⋯N2	0.80 (3)	2.35 (3)	2.688 (3)	106 (2)
N3—H3⋯N4	0.86 (3)	2.30 (3)	2.681 (3)	107 (2)
N5—H5⋯N6	0.84 (2)	2.25 (2)	2.670 (3)	111.1 (17)
O2—H2⋯O6^i^	0.91 (2)	1.65 (2)	2.549 (2)	169 (2)
O4—H4⋯O6^i^	0.91 (3)	1.66 (3)	2.5744 (19)	177 (3)
N1—H1⋯N6^ii^	0.80 (2)	2.55 (3)	3.224 (2)	143 (2)
N5—H5⋯O2^iii^	0.84 (2)	2.35 (2)	3.058 (2)	142.6 (19)
C5—H5*A*⋯O4^iv^	0.93	2.61	3.341 (3)	136
C17—H17⋯N2^iii^	0.93	2.60	3.330 (3)	136

Symmetry codes: (i) 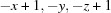; (ii) 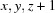; (iii) 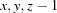; (iv) 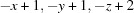.

**Table 2:** Experimental details

Crystal data	
Chemical formula	[Na_2_(C_6_H_5_N_2_O_2_)_2_(C_6_H_6_N_2_O_2_)_4_]
*M*_r_	872.73
Crystal system, space group	Triclinic, *P* 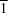
Temperature (K)	298
*a*, *b*, *c* (Å)	9.7997 (7), 10.0959 (7), 11.0401 (8)
α, β, γ (°)	96.618 (6), 102.741 (6), 113.902 (7)
*V* (Å^3^)	948.02 (13)
*Z*	1
Radiation type	Mo *K*α
μ (mm^−1^)	0.14
Crystal size (mm)	0.3 × 0.3 × 0.3

Data collection	
Diffractometer	Agilent Xcalibur Sapphire3
Absorption correction	Multi-scan (*CrysAlis PRO*; Agilent, 2013 ▸)
*T*_min_, *T*_max_	0.965, 1.000
No. of measured, independent and observed [*I* > 2σ(*I*)] reflections	12244, 5514, 3066
*R*_int_	0.032
(sin θ/λ)_max_ (Å^−1^)	0.703

Refinement	
*R*[*F* ^2^ > 2σ(*F* ^2^)], *wR*(*F* ^2^), *S*	0.064, 0.144, 0.96
No. of reflections	5521
No. of parameters	300
H-atom treatment	H atoms treated by a mixture of independent and constrained refinement
Δρ_max_, Δρ_min_ (e Å^−3^)	0.23, −0.23

Computer programs: *CrysAlis PRO* (Agilent, 2013 ▸), *SHELXT* (Sheldrick, 2015*a* ▸), *SHELXL2014* (Sheldrick, 2015*b* ▸), *OLEX2* (Dolomanov *et al.*, 2009 ▸).

## supplementary crystallographic information

### Crystal data

**Table:**

[Na_2_(C_6_H_5_N_2_O_2_)_2_(C_6_H_6_N_2_O_2_)_4_]	*Z* = 1
*M**_r_* = 872.73	*F*(000) = 452
Triclinic, *P*1	*D*_x_ = 1.529 Mg m^−^^3^
*a* = 9.7997 (7) Å	Mo *K*α radiation, λ = 0.71073 Å
*b* = 10.0959 (7) Å	Cell parameters from 2896 reflections
*c* = 11.0401 (8) Å	θ = 3.2–31.6°
α = 96.618 (6)°	µ = 0.14 mm^−^^1^
β = 102.741 (6)°	*T* = 298 K
γ = 113.902 (7)°	Block, clear colourless
*V* = 948.02 (13) Å^3^	0.3 × 0.3 × 0.3 mm

### Data collection

**Table:**

Agilent Xcalibur Sapphire3 diffractometer	5514 independent reflections
Radiation source: Enhance (Mo) X-ray Source	3066 reflections with *I* > 2σ(*I*)
Graphite monochromator	*R*_int_ = 0.032
Detector resolution: 16.1827 pixels mm^-1^	θ_max_ = 30.0°, θ_min_ = 3.0°
ω scans	*h* = −13→13
Absorption correction: multi-scan (CrysAlis PRO; Agilent, 2013)	*k* = −14→14
*T*_min_ = 0.965, *T*_max_ = 1.000	*l* = −15→15
12244 measured reflections	

### Refinement

**Table:**

Refinement on *F*^2^	0 restraints
Least-squares matrix: full	H atoms treated by a mixture of independent and constrained refinement
*R*[*F*^2^ > 2σ(*F*^2^)] = 0.064	*w* = 1/[σ^2^(*F*_o_^2^) + (0.0618*P*)^2^] where *P* = (*F*_o_^2^ + 2*F*_c_^2^)/3
*wR*(*F*^2^) = 0.144	(Δ/σ)_max_ < 0.001
*S* = 0.96	Δρ_max_ = 0.23 e Å^−^^3^
5521 reflections	Δρ_min_ = −0.23 e Å^−^^3^
300 parameters	

### Special details

**Table:**

Geometry. All esds (except the esd in the dihedral angle between two l.s. planes) are estimated using the full covariance matrix. The cell esds are taken into account individually in the estimation of esds in distances, angles and torsion angles; correlations between esds in cell parameters are only used when they are defined by crystal symmetry. An approximate (isotropic) treatment of cell esds is used for estimating esds involving l.s. planes.

### Fractional atomic coordinates and isotropic or equivalent isotropic displacement parameters (Å^2^)

**Table:**

	*x*	*y*	*z*	*U*_iso_*/*U*_eq_	
Na1	0.46101 (9)	0.10567 (8)	0.60562 (6)	0.0406 (2)	
O1	0.23043 (17)	0.04309 (17)	0.66132 (12)	0.0517 (4)	
O2	0.33418 (18)	0.01016 (16)	0.90258 (13)	0.0445 (4)	
H2	0.426 (3)	0.053 (3)	0.883 (2)	0.066 (8)*	
O3	0.56164 (19)	0.36282 (15)	0.66630 (13)	0.0555 (4)	
O4	0.74368 (18)	0.39266 (18)	0.90321 (14)	0.0516 (4)	
H4	0.681 (3)	0.293 (3)	0.876 (3)	0.107 (11)*	
O5	0.39508 (16)	0.03347 (14)	0.38677 (12)	0.0413 (3)	
O6	0.42798 (15)	−0.11024 (13)	0.17918 (12)	0.0374 (3)	
N1	0.2660 (2)	0.10347 (19)	0.87255 (16)	0.0397 (4)	
H1	0.250 (3)	0.145 (3)	0.930 (2)	0.075 (9)*	
N2	0.1263 (2)	0.28298 (18)	0.84096 (15)	0.0445 (4)	
N3	0.6408 (2)	0.45461 (19)	0.87873 (17)	0.0428 (4)	
H3	0.643 (3)	0.517 (3)	0.940 (2)	0.068 (8)*	
N4	0.4699 (2)	0.60614 (18)	0.85596 (16)	0.0454 (4)	
N5	0.34178 (18)	−0.03212 (17)	0.17342 (15)	0.0325 (4)	
H5	0.301 (2)	−0.018 (2)	0.104 (2)	0.049 (6)*	
N6	0.16908 (19)	0.11506 (18)	0.13287 (14)	0.0391 (4)	
C1	0.1203 (2)	0.19678 (19)	0.73700 (17)	0.0327 (4)	
C2	0.0281 (3)	0.1793 (3)	0.61794 (19)	0.0515 (6)	
H2A	0.0291	0.1203	0.5472	0.062*	
C3	−0.0661 (3)	0.2507 (3)	0.6045 (2)	0.0596 (6)	
H3A	−0.1316	0.2385	0.5248	0.072*	
C4	−0.0623 (2)	0.3393 (2)	0.7094 (2)	0.0483 (5)	
H4A	−0.1247	0.3889	0.7029	0.058*	
C5	0.0357 (3)	0.3532 (2)	0.8243 (2)	0.0522 (6)	
H5A	0.0399	0.4154	0.8954	0.063*	
C6	0.2119 (2)	0.1080 (2)	0.75329 (17)	0.0337 (4)	
C7	0.4699 (2)	0.52978 (19)	0.74923 (18)	0.0358 (4)	
C8	0.3960 (3)	0.5331 (3)	0.6301 (2)	0.0583 (6)	
H8	0.3977	0.4771	0.5579	0.070*	
C9	0.3190 (3)	0.6209 (3)	0.6188 (2)	0.0679 (7)	
H9	0.2696	0.6265	0.5387	0.082*	
C10	0.3159 (3)	0.6994 (2)	0.7263 (2)	0.0515 (6)	
H10	0.2635	0.7584	0.7214	0.062*	
C11	0.3925 (3)	0.6887 (2)	0.8421 (2)	0.0502 (6)	
H11	0.3905	0.7424	0.9155	0.060*	
C12	0.5611 (2)	0.44067 (19)	0.76097 (18)	0.0371 (4)	
C13	0.2335 (2)	0.11871 (18)	0.25376 (16)	0.0301 (4)	
C14	0.2116 (3)	0.1935 (3)	0.3547 (2)	0.0540 (6)	
H14	0.2580	0.1948	0.4382	0.065*	
C15	0.1199 (3)	0.2665 (3)	0.3297 (2)	0.0595 (6)	
H15	0.1045	0.3184	0.3964	0.071*	
C16	0.0524 (2)	0.2621 (2)	0.2072 (2)	0.0431 (5)	
H16	−0.0103	0.3101	0.1881	0.052*	
C17	0.0792 (2)	0.1845 (2)	0.11211 (19)	0.0465 (5)	
H17	0.0316	0.1803	0.0280	0.056*	
C18	0.3310 (2)	0.03654 (18)	0.27692 (16)	0.0299 (4)	

### Atomic displacement parameters (Å^2^)

**Table:**

	*U*^11^	*U*^22^	*U*^33^	*U*^12^	*U*^13^	*U*^23^
Na1	0.0567 (5)	0.0543 (5)	0.0255 (4)	0.0397 (4)	0.0115 (3)	0.0068 (3)
O1	0.0587 (9)	0.0844 (10)	0.0293 (8)	0.0518 (9)	0.0114 (7)	0.0031 (7)
O2	0.0571 (10)	0.0604 (9)	0.0423 (8)	0.0450 (8)	0.0217 (7)	0.0219 (7)
O3	0.0912 (12)	0.0558 (9)	0.0339 (8)	0.0507 (9)	0.0141 (8)	0.0025 (7)
O4	0.0543 (9)	0.0553 (9)	0.0494 (9)	0.0368 (8)	0.0044 (7)	0.0007 (7)
O5	0.0575 (9)	0.0562 (8)	0.0241 (7)	0.0395 (7)	0.0098 (6)	0.0101 (6)
O6	0.0486 (8)	0.0475 (7)	0.0333 (7)	0.0374 (7)	0.0128 (6)	0.0101 (6)
N1	0.0550 (11)	0.0544 (10)	0.0294 (9)	0.0410 (9)	0.0154 (8)	0.0121 (8)
N2	0.0551 (11)	0.0550 (10)	0.0325 (9)	0.0366 (9)	0.0089 (8)	0.0040 (8)
N3	0.0558 (11)	0.0469 (10)	0.0349 (10)	0.0347 (9)	0.0103 (8)	0.0035 (8)
N4	0.0634 (12)	0.0487 (9)	0.0362 (10)	0.0343 (9)	0.0198 (8)	0.0082 (8)
N5	0.0432 (9)	0.0423 (9)	0.0234 (8)	0.0307 (8)	0.0079 (7)	0.0079 (7)
N6	0.0462 (10)	0.0531 (10)	0.0313 (9)	0.0343 (8)	0.0124 (7)	0.0101 (7)
C1	0.0354 (10)	0.0400 (10)	0.0292 (10)	0.0206 (8)	0.0130 (8)	0.0101 (8)
C2	0.0690 (15)	0.0727 (14)	0.0288 (11)	0.0515 (13)	0.0081 (10)	0.0065 (10)
C3	0.0722 (16)	0.0826 (16)	0.0386 (13)	0.0562 (14)	0.0033 (11)	0.0097 (11)
C4	0.0554 (13)	0.0542 (12)	0.0515 (14)	0.0399 (11)	0.0139 (11)	0.0153 (10)
C5	0.0673 (15)	0.0583 (13)	0.0402 (12)	0.0424 (12)	0.0105 (11)	−0.0001 (10)
C6	0.0342 (10)	0.0436 (10)	0.0273 (9)	0.0216 (9)	0.0092 (8)	0.0061 (8)
C7	0.0418 (11)	0.0306 (9)	0.0348 (10)	0.0162 (8)	0.0118 (8)	0.0049 (8)
C8	0.0881 (18)	0.0653 (14)	0.0330 (12)	0.0537 (14)	0.0065 (11)	0.0004 (10)
C9	0.0954 (19)	0.0793 (16)	0.0420 (14)	0.0639 (16)	−0.0008 (13)	0.0063 (12)
C10	0.0547 (14)	0.0495 (12)	0.0598 (15)	0.0335 (11)	0.0152 (11)	0.0112 (11)
C11	0.0666 (15)	0.0522 (12)	0.0471 (13)	0.0377 (12)	0.0257 (11)	0.0072 (10)
C12	0.0485 (12)	0.0324 (9)	0.0336 (11)	0.0202 (9)	0.0144 (9)	0.0065 (8)
C13	0.0333 (10)	0.0323 (9)	0.0279 (9)	0.0171 (8)	0.0104 (7)	0.0067 (7)
C14	0.0792 (16)	0.0755 (15)	0.0311 (11)	0.0598 (14)	0.0132 (10)	0.0079 (10)
C15	0.0878 (18)	0.0793 (16)	0.0399 (13)	0.0650 (15)	0.0215 (12)	0.0066 (11)
C16	0.0480 (12)	0.0487 (11)	0.0483 (13)	0.0333 (10)	0.0191 (10)	0.0143 (10)
C17	0.0543 (13)	0.0663 (13)	0.0350 (11)	0.0420 (11)	0.0115 (10)	0.0161 (10)
C18	0.0342 (10)	0.0327 (9)	0.0270 (9)	0.0183 (8)	0.0101 (7)	0.0063 (7)

### Geometric parameters (Å, º)

**Table:**

Na1—O5	2.3044 (14)	C1—C2	1.367 (3)
Na1—O3	2.3225 (15)	C1—C6	1.503 (2)
Na1—O1	2.3300 (16)	C2—C3	1.377 (3)
Na1—O5^i^	2.3558 (14)	C2—H2A	0.9300
Na1—O6^i^	2.3716 (14)	C3—C4	1.363 (3)
Na1—Na1^i^	3.3964 (13)	C3—H3A	0.9300
O1—C6	1.232 (2)	C4—C5	1.365 (3)
O2—N1	1.388 (2)	C4—H4A	0.9300
O2—H2	0.91 (2)	C5—H5A	0.9300
O3—C12	1.235 (2)	C7—C8	1.366 (3)
O4—N3	1.383 (2)	C7—C12	1.501 (3)
O4—H4	0.91 (3)	C8—C9	1.378 (3)
O5—C18	1.247 (2)	C8—H8	0.9300
O5—Na1^i^	2.3558 (14)	C9—C10	1.362 (3)
O6—N5	1.3666 (18)	C9—H9	0.9300
O6—Na1^i^	2.3716 (14)	C10—C11	1.372 (3)
N1—C6	1.320 (2)	C10—H10	0.9300
N1—H1	0.80 (2)	C11—H11	0.9300
N2—C1	1.333 (2)	C13—C14	1.379 (3)
N2—C5	1.339 (2)	C13—C18	1.500 (2)
N3—C12	1.319 (3)	C14—C15	1.379 (3)
N3—H3	0.86 (2)	C14—H14	0.9300
N4—C7	1.332 (2)	C15—C16	1.355 (3)
N4—C11	1.336 (2)	C15—H15	0.9300
N5—C18	1.314 (2)	C16—C17	1.372 (3)
N5—H5	0.84 (2)	C16—H16	0.9300
N6—C17	1.330 (2)	C17—H17	0.9300
N6—C13	1.334 (2)		
			
O5—Na1—O3	109.39 (6)	N2—C5—C4	124.16 (19)
O5—Na1—O1	107.77 (5)	N2—C5—H5A	117.9
O3—Na1—O1	98.10 (6)	C4—C5—H5A	117.9
O5—Na1—O5^i^	86.43 (5)	O1—C6—N1	123.81 (17)
O3—Na1—O5^i^	126.64 (6)	O1—C6—C1	121.78 (17)
O1—Na1—O5^i^	125.87 (6)	N1—C6—C1	114.39 (16)
O5—Na1—O6^i^	156.53 (5)	N4—C7—C8	123.26 (18)
O3—Na1—O6^i^	87.61 (5)	N4—C7—C12	118.11 (17)
O1—Na1—O6^i^	84.80 (5)	C8—C7—C12	118.58 (17)
O5^i^—Na1—O6^i^	70.26 (5)	C7—C8—C9	118.8 (2)
C6—O1—Na1	127.13 (13)	C7—C8—H8	120.6
N1—O2—H2	102.4 (15)	C9—C8—H8	120.6
C12—O3—Na1	129.55 (13)	C10—C9—C8	119.3 (2)
N3—O4—H4	103.9 (19)	C10—C9—H9	120.4
C18—O5—Na1	151.93 (12)	C8—C9—H9	120.4
C18—O5—Na1^i^	114.42 (11)	C9—C10—C11	118.0 (2)
Na1—O5—Na1^i^	93.57 (5)	C9—C10—H10	121.0
N5—O6—Na1^i^	110.28 (9)	C11—C10—H10	121.0
C6—N1—O2	121.64 (15)	N4—C11—C10	124.06 (19)
C6—N1—H1	121.1 (19)	N4—C11—H11	118.0
O2—N1—H1	116.7 (19)	C10—C11—H11	118.0
C1—N2—C5	116.66 (17)	O3—C12—N3	123.70 (18)
C12—N3—O4	121.18 (16)	O3—C12—C7	121.61 (18)
C12—N3—H3	119.5 (17)	N3—C12—C7	114.69 (16)
O4—N3—H3	118.2 (17)	N6—C13—C14	122.09 (17)
C7—N4—C11	116.62 (18)	N6—C13—C18	117.42 (15)
C18—N5—O6	121.82 (15)	C14—C13—C18	120.49 (17)
C18—N5—H5	116.6 (14)	C13—C14—C15	118.87 (19)
O6—N5—H5	121.3 (14)	C13—C14—H14	120.6
C17—N6—C13	117.46 (16)	C15—C14—H14	120.6
N2—C1—C2	123.00 (17)	C16—C15—C14	119.55 (19)
N2—C1—C6	118.12 (16)	C16—C15—H15	120.2
C2—C1—C6	118.77 (16)	C14—C15—H15	120.2
C1—C2—C3	118.83 (19)	C15—C16—C17	118.02 (18)
C1—C2—H2A	120.6	C15—C16—H16	121.0
C3—C2—H2A	120.6	C17—C16—H16	121.0
C4—C3—C2	119.3 (2)	N6—C17—C16	124.00 (19)
C4—C3—H3A	120.3	N6—C17—H17	118.0
C2—C3—H3A	120.3	C16—C17—H17	118.0
C3—C4—C5	117.99 (19)	O5—C18—N5	123.20 (16)
C3—C4—H4A	121.0	O5—C18—C13	121.78 (15)
C5—C4—H4A	121.0	N5—C18—C13	115.02 (15)
			
Na1^i^—O6—N5—C18	1.8 (2)	Na1—O3—C12—C7	−113.54 (18)
C5—N2—C1—C2	0.4 (3)	O4—N3—C12—O3	6.3 (3)
C5—N2—C1—C6	−175.88 (18)	O4—N3—C12—C7	−172.72 (16)
N2—C1—C2—C3	−1.8 (3)	N4—C7—C12—O3	177.27 (19)
C6—C1—C2—C3	174.5 (2)	C8—C7—C12—O3	−5.1 (3)
C1—C2—C3—C4	1.6 (4)	N4—C7—C12—N3	−3.7 (3)
C2—C3—C4—C5	−0.1 (4)	C8—C7—C12—N3	173.90 (19)
C1—N2—C5—C4	1.2 (3)	C17—N6—C13—C14	−1.3 (3)
C3—C4—C5—N2	−1.4 (4)	C17—N6—C13—C18	178.22 (17)
Na1—O1—C6—N1	−72.4 (2)	N6—C13—C14—C15	0.2 (3)
Na1—O1—C6—C1	109.31 (18)	C18—C13—C14—C15	−179.3 (2)
O2—N1—C6—O1	−7.4 (3)	C13—C14—C15—C16	0.6 (4)
O2—N1—C6—C1	170.99 (16)	C14—C15—C16—C17	−0.3 (4)
N2—C1—C6—O1	−169.34 (18)	C13—N6—C17—C16	1.7 (3)
C2—C1—C6—O1	14.2 (3)	C15—C16—C17—N6	−0.9 (3)
N2—C1—C6—N1	12.2 (3)	Na1—O5—C18—N5	175.36 (18)
C2—C1—C6—N1	−164.23 (19)	Na1^i^—O5—C18—N5	0.2 (2)
C11—N4—C7—C8	0.0 (3)	Na1—O5—C18—C13	−5.3 (4)
C11—N4—C7—C12	177.53 (17)	Na1^i^—O5—C18—C13	179.48 (12)
N4—C7—C8—C9	0.8 (4)	O6—N5—C18—O5	−1.4 (3)
C12—C7—C8—C9	−176.6 (2)	O6—N5—C18—C13	179.24 (14)
C7—C8—C9—C10	−1.3 (4)	N6—C13—C18—O5	−179.72 (17)
C8—C9—C10—C11	0.9 (4)	C14—C13—C18—O5	−0.2 (3)
C7—N4—C11—C10	−0.5 (3)	N6—C13—C18—N5	−0.4 (2)
C9—C10—C11—N4	0.0 (4)	C14—C13—C18—N5	179.15 (19)
Na1—O3—C12—N3	67.5 (3)		

Symmetry code: (i) −*x*+1, −*y*, −*z*+1.

### Hydrogen-bond geometry (Å, º)

**Table:**

*D*—H···*A*	*D*—H	H···*A*	*D*···*A*	*D*—H···*A*
N1—H1···N2	0.80 (3)	2.35 (3)	2.688 (3)	106 (2)
N3—H3···N4	0.86 (3)	2.30 (3)	2.681 (3)	107 (2)
N5—H5···N6	0.84 (2)	2.25 (2)	2.670 (3)	111.1 (17)
O2—H2···O6^i^	0.91 (2)	1.65 (2)	2.549 (2)	169 (2)
O4—H4···O6^i^	0.91 (3)	1.66 (3)	2.5744 (19)	177 (3)
N1—H1···N6^ii^	0.80 (2)	2.55 (3)	3.224 (2)	143 (2)
N5—H5···O2^iii^	0.84 (2)	2.35 (2)	3.058 (2)	142.6 (19)
C5—H5*A*···O4^iv^	0.93	2.61	3.341 (3)	136
C17—H17···N2^iii^	0.93	2.60	3.330 (3)	136

Symmetry codes: (i) −*x*+1, −*y*, −*z*+1; (ii) *x*, *y*, *z*+1; (iii) *x*, *y*, *z*−1; (iv) −*x*+1, −*y*+1, −*z*+2.
